# Epidemiological evaluation of patient compliance regarding oral health and hygiene during the COVID-19 period

**DOI:** 10.31744/einstein_journal/2023AO0195

**Published:** 2023-09-15

**Authors:** Antonia Sinesi, Valentino Natoli, Cinzia Casu, Savino Cefola, Ruggiero Damato, Roberta Grassi, Gianna Maria Nardi

**Affiliations:** 1 Canosa di Puglia Italy Registered Dental Hygienist, Canosa di Puglia, Italy.; 2 Department of Dentistry European University of Madrid Madrid Spain Department of Dentistry, European University of Madrid, Madrid, Spain.; 3 Department of Surgical Science Oral Biotechnology Laboratory University of Cagliari Cagliari Italy Department of Surgical Science, Oral Biotechnology Laboratory, University of Cagliari, Cagliari, Italy.; 4 Barletta Italy Doctor of Dental Science, Private Dental Practice, Barletta, Italy.; 5 Barletta Italy Registered Dental Hygienist, Barletta, Italy.; 6 Department of Biomedical Sciences University of Sassari Sassari Italy Department of Biomedical Sciences, University of Sassari, Sassari, Italy.; 7 Department of Odontostomatological and Maxillofacial Sciences Sapienza Università di Roma Roma Italy Department of Odontostomatological and Maxillofacial Sciences, Sapienza Università di Roma, Roma, Italy.

**Keywords:** Oral hygiene, Oral health, Patient compliance, Epidemiologic studies, COVID-19, Coronavirus infections, Surveys and questionnaires

## Abstract

**Objective:**

This work aimed to report values regarding oral hygiene habits at home during the pandemic and compare the difference with previous oral hygiene habits.

**Methods:**

This study was performed anonymously and had patients answer an online questionnaire. The questionnaire consisted of 11 closed-answer questions (except 1) concerning oral hygiene habits at home at the time of COVID-19 and participant characteristics, including gender and age. A total of 1,136 questionnaires were filled in via a link sent by email to the patients.

**Results:**

Among the 1,136 patients examined, 32.4% (372) were male and 67.6% (775) were female, and almost half (47.9%) of the patients who answered the questionnaire were aged 18-40 years. Patients who participated in our survey were asked questions related to dental and tongue hygiene. The answers showed that although they had more time during the pandemic, they brushed their teeth less and did not brush their tongues regularly.

**Conclusion:**

Patients with periodontal problems usually have less time for brushing and do not have good plaque control. The questionnaire showed the opposite, such that the patient’s compliance and motivation matters more than time. Thus, dentists and dental hygienists must encourage and make patients understand the importance of oral hygiene at home.

## In Brief

This work aimed to report values on oral hygiene habits at home during the pandemic and compare the differences between previous oral hygiene habits. A total of 1,136 questionnaires were filled in via a link sent through email to the patients. The answers show that although they had more time during the pandemic, they spent less time on dental and tongue hygiene.

## Highlights

Dental and tongue hygiene are key to maintaining systemic health.

The questionnaire was sent by e-mail to 1,136 male and female patients throughout Italy.

Demonstrate and understand whether patients during the pandemic cleaned and spent time cleaning and maintaining their teeth and tongue hygiene.

The questionnaire testifies to the fact that it is not the time but the patient’s compliance and motivation that makes the difference.

## Graphical abstract

**Figure:**
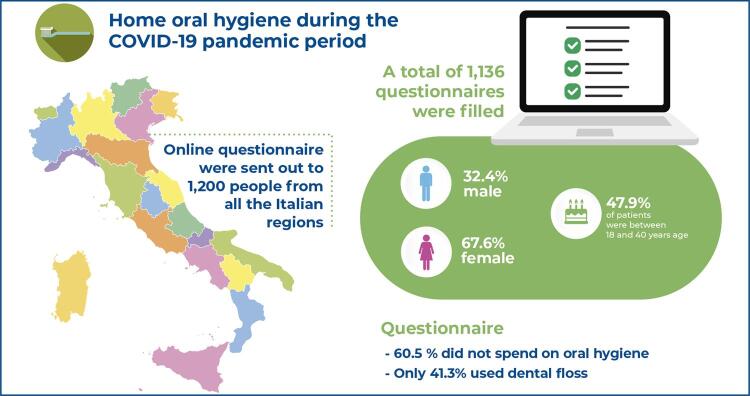

## INTRODUCTION

Oral hygiene techniques must be performed not only to maintain oral health, but also systemic health.^(1,2)^ Home oral hygiene is important for periodontal health.^(3)^ However, the frequency and type of oral hygiene maneuvers change considerably in various geographical areas. Differences in oral hygiene habits have been shown to be related to culture and geographic region. A recent study reported that 73-83% of schoolchildren in Norway, Germany, Sweden, Denmark, and Austria brushed their teeth twice daily; meanwhile, toothbrushing frequency was reported by only 19-46% of patients in Lithuania, Saudi Arabia, and Japan.^(4)^ In Saudi Arabia, the frequency of dental hygiene is higher in women than in men.^(5)^ In rural Indian areas, more than 40% of the population studied did not consider brushing to be essential in preventing oral diseases; moreover, a good portion of the population believed that professional oral procedures can deteriorate the teeth.^(6)^ A survey performed on 300 people compared the habits of an Indian population with that of those from the Dominican Republic. According to this study, on an equal socio-economic basis, 55% of the interviewed Indian population used a toothbrush and approximately 58% used it at least twice a day, in contrast to those from the Dominican Republic, in which 94% use their toothbrush at least twice a day.^(7)^ A study on a large sample of population from Kuwait (more than 1,950 participants) showed that approximately 62% brushed their teeth at least twice a day, while only 11% used dental floss and 33% used milwaki.^(4)^

Returning to a European context, a study on 1,200 participants regarding oral hygiene habits revealed that 97% of Portuguese people used their toothbrush daily: 77% of women brush their teeth twice a day, but only 68% of men do.^(8)^ Studies in a Polish population reported that approximately 80% performed oral hygiene once or twice a day^(9)^ and 30% performed oral hygiene only once a day or less.^(10)^ However, few studies on home hygiene habits on a population in conditions of isolation or rapid economic difficulty, such as the conditions brought about by the COVID-19 pandemic, are available. An Icelandic study evaluated the habits of 4,100 people between 2007 and 2009, which are the years of the country’s severe economic crisis. The results showed that previously, 96.8% of the participants performed daily oral hygiene and 40% used dental floss; however, during the crisis, the percentage increased to 97.4% and 43% respectively.^(11)^ A significantly smaller study (300 participants) in Lithuania, which was performed during a period of economic reforms in the country, showed that the 35-44 year-old age group performed dental cleaning twice a day in 35% of cases, while in the 65-74 year-old age group, only 21% performed.^(12)^ A previous study administered questionnaires in three different Italian universities (Palermo, Milan, Rome) and did not obtain particularly encouraging results especially on the use of dental floss among the Italian population.^(13)^ Furthermore, epidemiological studies of this type seem to be aimed mainly at particular categories of patients (children, elderly, patients with particular systemic pathologies, etc.). Few data are also collected on the duration of hygiene maneuvers, lingual cleaning, and differences between a certain historical period and another. Thus, this study aimed to report the values on home oral hygiene habits during the pandemic and their difference in comparison with previous oral hygiene habits.

In January 2020, a health emergency was declared due to COVID-19. Consuequently, the governments of several countries, including Italy, have taken strict measures to prevent contagion in the face of this pandemic. Thus, restrictions on travel, social life, medical and dental check-ups have had a strong impact on the oral and periodontal health of the population.^(14)^

## OBJECTIVE

Demonstrate and understand whether patients during the pandemic cleaned or spent time cleaning and maintaining their teeth and tongue hygiene.

## METHODS

This study was performed anonymously and had patients answer an online questionnaire. The questions were sent via email to 1,200 people from all regions of Italy. The questions asked too patients were created in Google Form was used to create the surveys.

The questionnaire consisted of 11 closed-answer questions (except 1) concerning oral hygiene habits at home at the time of COVID-19 and participant characteristics, including gender and age. The questions included three or four answer options (Appendix 1).

A total of 1,136 questionnaires were filled in (questionnaires were sent out to 1,200 people) via a link sent by email to the patients. All respondents were included, and none were excluded.

## RESULTS

Among the 1,136 patients examined, 32.4% (372) were male and 67.6% (775) were female. Almost half (47.9%) of the patients who answered the questionnaire were aged 18-40 years.

**Figure 1:**
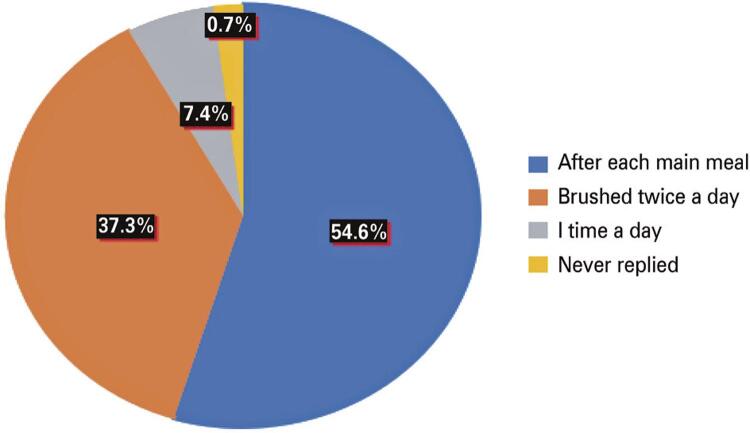
How many times a day did you brush your teeth before the COVID-19 emergency?

The replies received were analyzed, and graphs were constructed subsequently to examine and relate the various data. Regarding the question, “How many times a day did you brush your teeth before the COVID-19 emergency?” 54.6% of the participants indicated that they brushed their teeth after each main meal; 37.3% brushed twice a day; 7.4% brushed only once a day; and 0.7% did not answer (Figure 1). Question number 4 asked about the frequency of daily tooth brushing today.

**Figure 2:**
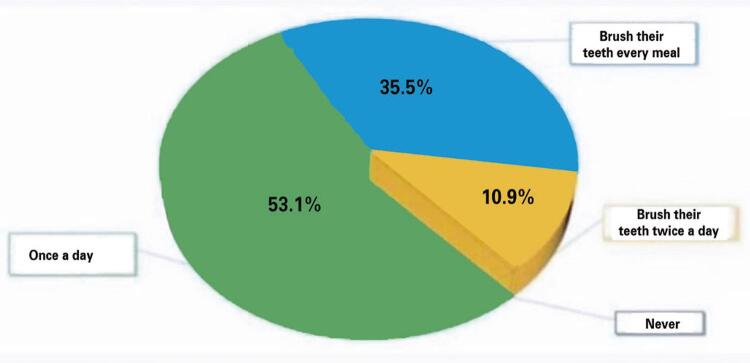
How many times do you brush your teeth per day now?

**Figure 3:**
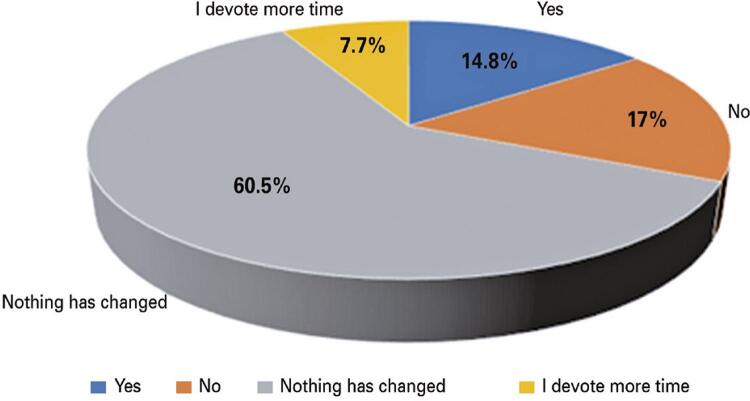
Do you plan to spend more time on daily oral hygiene during this time?

**Figure 4:**
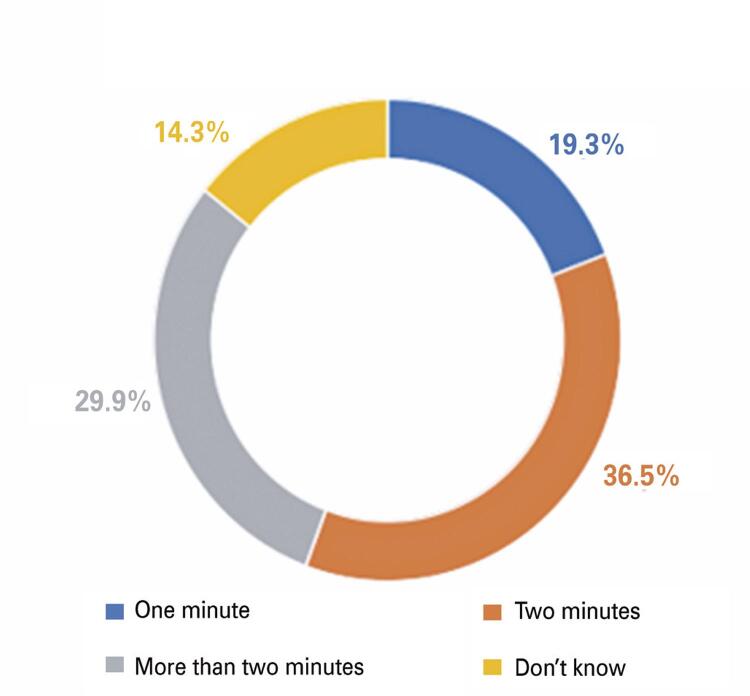
How long did it take you to brush your teeth before the COVID-19 pandemic?

**Figure 5:**
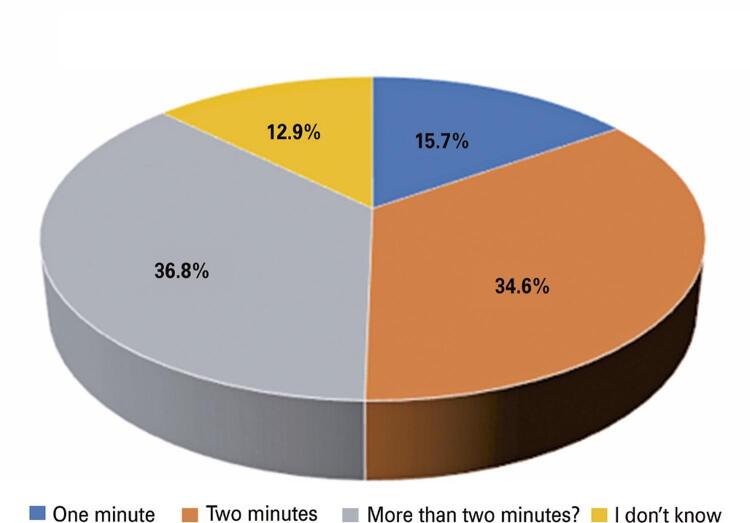
How much time do you spend brushing your teeth these days?

The answers showed that 35.5% brushed their teeth after every meal, 10.9% brushed their teeth twice a day, and 53.1% brushed once a day (Figure 2). Regarding the question, “Do you plan to spend more time on daily oral hygiene during this time?” 60.5% replied that nothing has changed, 14.8% replied YES, 17% replied NO, and only 7.7% spent more time (Figure 3). Subsequently, they were asked how long it took to brush their teeth before the COVID-19 pandemic. A total of 36.5% answered 2 minutes, 29.9% more than 2 minutes, 19.3% 1 minutes, and finally 14.3% answered that they did not know how long it took to brush their teeth (Figure 4). The next question is regarding the duration of their brushing during the pandemic. A total of 36.8% brushed their teeth for more than 2 minutes, 34.6% for 2 minutes, 15.7% for only 1 minutes, and 12.9% were not able to quantify the time (Figure 5).

**Figure 6:**
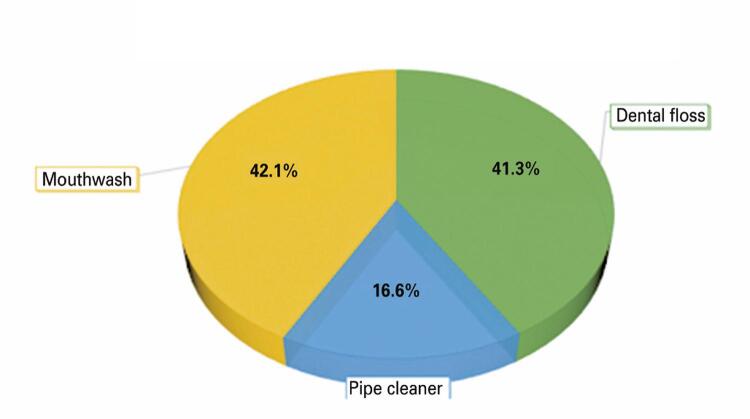
Do you use any other daily oral care products? If yes, which ones?

**Figure 7:**
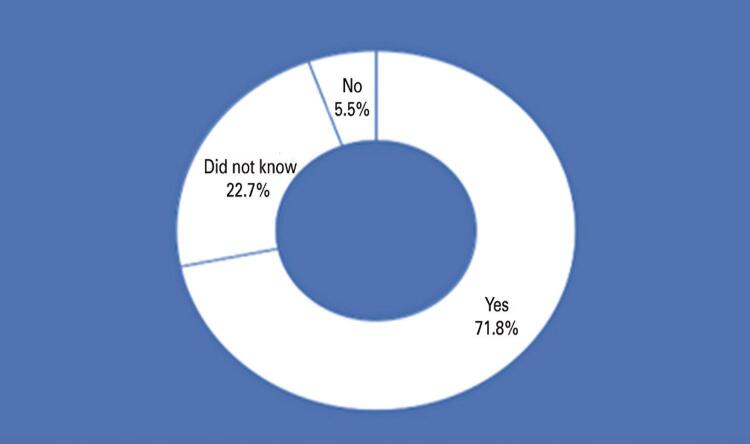
Do you think it is important to brush your tongue?

Regarding the use of other oral hygiene devices, 42.1% of the participants replied that they used mouthwash, 41.3% used floss, and 16.6% only used a toothbrush (Figure 6). The last three questions concerned the cleaning of the tongue. Regarding the question, “Do you think it is important to brush the tongue?” 71.8% answered YES, 22.7% did not know, and only 5.5% answered NO (Figure 7). Subsequently, they were asked if they brushed their tongue regularly, and only 29% answered YES, 33.5% answered sometimes, and 29% answered NO. The last question was regarding the kind of brush they used to clean the tongue, and 66.1% used a toothbrush, 22% did not use anything, and 11.9% used a tongue cleaner.

## DISCUSSION

This cognitive survey, which aimed to report the values of oral hygiene habits at home during the pandemic period and its difference in comparison with oral hygiene habits before the pandemic, gathered a good number of responses.

An Icelandic study, which assessed the habits of 4,100 people between 2007 and 2009, found that even though people had more time during lockdown, they did not spend more time on oral hygiene at home. Moreover, even though they recognize the importance of it, they did not brush their tongues regularly. In fact, 60.5% of the patients who answered our questionnaire admitted that they did not spend more time on oral hygiene during the period of home confinement, and only 41.3% used dental floss, against respectively 97.4% and 43% in the Icelandic study.^(11)^

However, it must be considered that the number of participants in the Icelandic study was over 4,100 people, while our survey only included 1,136 people. Thus, the comparison could also be affected by this numerical variability.

If data relating to oral hygiene habits before the pandemic in the literature were analyzed, we can see a certain heterogeneity between the various studies, both for the geographic area concerned, number of survey participants, and methods of questionnaires proposed.

A study, which analyzed the relationship between dental health behavior, oral hygiene, and periodontal status of 93 dental students in the United Arab Emirates, found that only 56% of the participating students flossed regularly. This is interesting to note since dental students are supposed to have a sufficient important knowledge on oral hygiene.^(15)^ The percentage of participants who used dental floss is much more in line with our data, which was collected during the pandemic period, compared with that in the Icelandic study.

However, another study of 314 students from Kuwait University in Jordan reported the opposite.^(16)^ Furthermore, an Australian study which examined adult oral hygiene behaviors (tooth brushing, mouthwash and flossing) found that females used dental floss more frequently than males, and that people with high socio-economic status brushed their teeth more often than people with low socio-economic status. The percentage of people who brushed their teeth daily ranged from 98% (women) to 94% (men); however, that of flossing was much lower: 62% (women), 48% (men). These data are close to the values we obtained during the pandemic period.^(17)^ The percentage data relating to the last three studies cited, which were performed not in periods of isolation, showed that, regardless of the different geographical locations and size of the population sample analyzed, the attention to oral hygiene even before the pre-pandemic period did not change.

Prevention remains the fundamental goal to facilitate the maintenance of the teeth and supporting structures.^(18)^ Oral health education is a fundamental part of dental health services and has been imparted in schools, universities, and facilities for the elderly. Health promotion is not something done on or for people, but with people, both as individuals and as a group.^(19)^ The long-term success of various dental treatments also implies that patients must be able to perform effective oral hygiene at home. In this sense, patients are required to play their part and discuss with the clinician in order to develop a home strategy based on the choice of devices indicated for specific characteristics and needs evaluated on the basis of learning skills.^(20)^

The available home devices are currently varied, with products adapted to the characteristics of the uniqueness of the oral cavity of each individual. As already mentioned, the choice of home hygiene devices must be made by assessing the patient’s compliance and home capabilities, as the effectiveness of each product is a function of correct use.^(21)^ Nevertheless, devices that are more familiar to and easier to use for the patient must be chosen.

Obviously, encouraging patient compliance is another indispensable requirement, since good oral hygiene at home is the result of scrupulous and repeated procedures, devoting the appropriate time to carry out all the necessary maneuvers in order to clean and maintain the oral structures in the best possible way.

In addition, the patient’s ability to correctly perform the recommended home oral hygiene techniques must be evaluated through regular checks of plaque accumulation during professional oral hygiene sessions, preferably with the help of revealing substances such as erythrosin-based solutions.^(22)^

Reagrding the manual toothbrush techniques, there are no precise indications; however, it is certainly advisable to give precise instructions to the patient, opting in any case for techniques that will not be traumatic for gum and peri-implant tissues, such as the modified Bass, Stillmann or roller technique. If electric toothbrushes are preferred, devices with a pulsating and rotating motion, equipped with round heads with medium or soft bristles and a simple design, are recommended.^(23)^

The survey highlighted some interesting aspects, such as the fact that the majority of the sample is aged 18-40 years. Moreover, regarding the question, “Do you plan to spend more time on daily oral hygiene during this time?” the most supported response was that nothing changed. This answer shows the psychological effects of the pandemic and how it also affected people’s oral hygiene maintenance.

Tension, restlessness, laziness, and neglect are all consequences of the pandemic.^(24)^ Comparing questions 3 and 4, a significant finding emerged: during the pandemic, people brushed their teeth less. In fact, 53.1% of the participants brushed their teeth only once a day compared to before the pandemic. This data shows us that patient compliance makes the difference and not time.

Compliance can only be achieved by motivating the patient. Patient motivation is essential for the results of therapies to be effective. One of the goals of all dentists and dental hygienists should be to incentivize and motivate all patients to perform oral hygiene techniques at home. Only intrinsic motivation drives change.^(25)^

Patients who responded to the survey were also given questions related to tongue hygiene. The keratinized and rough surface of the tongue may provide the necessary conditions for bacterial colonization, many of which produce volatile sulphur compounds responsible for halitosis.^(26)^ Adequate hygiene can reduce biofilm on the tongue.^(27)^ The elimination of biofilm on the surface of the tongue can be performed using specific products, such tongue brushes and scrapers.^(28)^

From the answers given by patients, it can be seen that people know the importance of brushing the tongue, but they do not always use a toothbrush. As evidenced by a study conducted in Japan where the goal was to study tongue cleaning habits using toothbrushes among patients in a hospital, it was deduced that only 18.4% of participants cleaned their tongues daily.^(29)^ Kishi et al.^(30)^ conducted a questionnaire survey about the tongue cleaning habits of 479 participants and reported that 37.0% of all participants replied that they practiced tongue cleaning.

In this study, patients recognized the importance of tongue cleaning (71.8%), but only 28.2% cleaned it regularly. This shows that the problem does not only concern the knowledge of the practice of lingual hygiene, but of the habit of carrying it out.

This study and the two Japanese studies showed that a low percentage of the population cleans their tongues, which shows that, regardless of cultural or geopolitical differences, there is no real habit of tongue hygiene. Thus, it is necessary to motivate patients to practice tongue hygiene.

## CONCLUSION

Patients with periodontal and gum problems usually do not have time to brush or good plaque control. However, the results of this questionnaire showed that this is not true as due to the pandemic, people have time. Nevertheless, patient compliance and motivation matters.

The primary role of every health worker is to promote prevention in health education, especially primary prevention, through effective communication. Most oral diseases are preventable. The dentist and dental hygienist must aim to identify patients at risk and help them achieve optimal oral and general health to improve their overall quality of life. The dentist and dental hygienist must strive to empower each patient and should inspire them.

## Appendix 1

The following questions were asked to patients


