# Supplementary material for: Epidemiological evaluation of patient compliance regarding oral health and hygiene during the COVID-19 period
Source: Einstein (Sao Paulo). 2023 Sep 15;21:eAO0195. doi: 10.31744/einstein_journal/2023AO0195 (PMC10519668; doi:10.31744/einstein_journal/2023AO0195)
Supplement: Supplementary file 1 [file 2317-6385-eins-21-eAO0195-suppl01.pdf]

Appendix 1. The following questions were asked to patients

1) Gender
   a) male
   b) female
2) How old are you?
   a) between 18 and 40 years of age
   b) over 40 and up to 50 years of age
   c) over 50 and up to 60 years of age
   d) over 60 years of age
   e) under 18 years of age
3) How many times a day did you brush your teeth before the COVID-19 emergency?
   a) after each main meal
   b) brushed twice a day
   c) one time a day
   d) never replied
4) How many times do you brush your teeth per day now?
   a) after each main meal
   b) two times a day
   c) one time a day
   d) never
5) Do you plan to spend more time on daily oral hygiene during this time?
   a) yes
   b) no
   c) nothing has changed
   d) I devote more time
6) How long did it take for you to brush your teeth before the COVID-19 pandemic?
   a) one minute
   b) two minutes
   c) more than two minutes
   d) don't know
7) How much time do you spend brushing your teeth these days?
   a) one minute 15.7%
   b) two minutes 34.6%
   c) more than two minutes 36.8%
   d) I don't know 12.9%
8) Do you use any other daily oral care products? If yes, which ones?
   a) dental floss
   b) pipe cleaner
   c) mouthwash
9) Do you think it is important to brush your tongue?
   a) yes
   b) I don't know
   c) no
10) Do you brush your tongue regularly?
   a) yes
   b) no
   c) sometimes
   d) rarely
11) What do you use to brush your tongue?
   a) nothing
   b) toothbrush
   c) tongue cleaner
